# How effective are interventions to reduce attacks on people from large carnivores? A systematic review protocol

**DOI:** 10.1186/s13750-024-00337-2

**Published:** 2024-05-17

**Authors:** Ann Eklund, Jens Frank, José Vicente López Bao

**Affiliations:** 1 Grimsö Wildlife Research Station, Department of Ecology, Swedish University of Agricultural Sciences, 739 93 Riddarhyttan, Sweden; 2 Biodiversity Research Institute, CSIC, Principality of Asturias, Oviedo University, Mieres, Spain

**Keywords:** Carnivore attack, Human-carnivore impact, Mitigation, Large carnivores, Systematic review

## Abstract

**Background:**

Instances of attacks from large carnivores that lead to human injury or death are increasingly reported worldwide. Ensuring human safety when people and carnivores co-occur is central to minimizing human suffering but is also essential to support sustainable carnivore conservation. Various interventions are available intended to alter either the behavior of large carnivores or people, in order to reduce the likelihood of a risky encounter and an attack. Collated evidence on best practices is still lacking, and this protocol outlines a systematic review of evidence for intervention effectiveness to reduce the risk or severity of direct attacks on humans by large carnivores. Specifically, the review seeks to answer the question: How effective are evaluated interventions in reducing large carnivore attacks on people?

**Methods:**

The bibliographic databases Zoological Record, BIOSIS Citation Index, and Scopus will be searched using a predefined search string. Grey literature will be requested through professional networks, contacts with relevant organizations, and searching selected websites. All returned titles and abstracts will be manually screened using Rayyan.ai. For inclusion, studies should describe the Population, Intervention, Comparator, and Outcome (PICO) of the review research question and be written in English, Spanish, or Swedish. Review papers will be excluded. All records of data coding and extraction are documented in a purposely developed, and priorly piloted, data sheet. Critical appraisal of study validity will be done according to the Collaboration for Environmental Evidence Critical Appraisal Tool prototype version 0.3. Review outcomes will be synthesized in a narrative, and if possible, a quantitative synthesis. The narrative synthesis will describe in text the carnivore population (species, location), context (target object, intervention model), as well as the design and reported results of each study. The quantitative synthesis will include a summary statistic, preferably logarithmic risk ratio, calculated for each original study. A forest plot will be created to visualize study outcomes, as well as judgments of critical appraisal. Provided that enough data is available and that it complies with its assumptions, a meta-regression analysis will be undertaken using metafor package for R software.

**Supplementary Information:**

The online version contains supplementary material available at 10.1186/s13750-024-00337-2.

## Background

Wildlife conservation efforts intended to support self-sustainable populations of wildlife often rely on land sharing approaches, where people and wildlife share multiuse landscapes [8, 13, 25, 43]. This applies to conservation and management of large carnivores (i.e., species in the order carnivora with a body mass of > 15 kg, [45]). Their viability cannot be sustained within the limited boundaries of protected areas in human-dominated landscapes [10], which are often too small considering the large spatial requirements of these species. The majority of protected areas in Europe, for example, are too small to sustain viable large carnivore populations [46] and, consequently, large carnivores persist in landscapes with varying levels of human activities and land transformation [10, 33]. This pattern can be observed around the globe for different species and contexts [9]. In India, an increasing number of tigers are found outside reserves [27]. On the other hand, increasing human populations means more people in more places, including protected or more remote areas (e.g., recreational activities [42]). As a consequence, with more large carnivores persisting in human-dominated landscapes, and more people everywhere, it is expected an increasing number of interactions between large carnivores and humans, and more risk-enhancing human behaviors that can increase the likelihood of risky encounters with wildlife.

Sharing the landscape can lead to more positive and beneficial interactions between humans and wildlife, but may also lead to interactions of a more negative kind [22, 36]. Negative interactions between wildlife and people can be indirect, such as when wild animals are involved in vehicle accidents (e.g., [12]) or through the spread of zoonotic disease (e.g., [29]). Wildlife can also cause more direct threats to human health, for instance if wild animals attack and physically injure or kill a person [36]. Instances of attacks from large carnivores that lead to human injury or death are increasingly reported worldwide [7]. Increases may reflect actual numbers of incidents, but could likewise reflect an increasing research interest or report bias with regards to wildlife interactions [36]. Syntheses of attack reports often focus on specific species or geographical regions, and the total number of incidents, involving all carnivore species, is likely difficult to estimate. Bombieri et al. [7] identify 5 440 large carnivore attacks between 1950 and 2019. This number is, however, based on data from a limited geographical range, and is likely an underestimate. Species specific worldwide syntheses have for example been made for brown bears (*Ursus arctos*), with 664 attacks recorded between 2000 and 2015 [6], or wolves (*Canis lupus*), with 489 attacks recorded between 2002 and 2020, of which 380 were rabid attacks [32]. Lions, leopards, and tigers (*Panthera* species) are also regionally reported as causing human injury or death (e.g., [1, 40]) and coyotes (*Canis latrans*) are reported to cause mainly bite wounds [51]. The context of wildlife attacks on people includes situations of human leisure or livelihood activities [6, 7] in forests, farmlands, urban settings, or even in homes [1, 6, 51].

Ensuring human safety when people and carnivores co-occur is central to minimizing human trauma and suffering (e.g., [11, 17, 44]). It is also essential to support sustainable conservation efforts, as increasing human health and safety concerns would likely compromise the support for future carnivore populations [48]. To avoid attacks on humans caused by large carnivores, various interventions are available intended to alter the behavior of the wild animals, or the behavior of humans near wildlife [51], or both. Interventions intended to temporarily alter animal behavior include, but are not limited to, more technical interventions such as scaring devices, deterrents, barriers, and fences (e.g., [14, 19, 26, 28]). There are also practical interventions which are intended to alter animal behavior long term, including efforts of aversive conditioning or relocation of the animals (e.g., [2, 3]). Interventions intended to alter the behavior of people around wildlife include information campaigns or experiential education about how to act near wildlife (e.g., [5, 47]).

Considering human safety and wellbeing, large carnivore conservation and a sound allocation of funding and the continuous development of best practices, it is important to collate evidence of intervention effectiveness and identify potential knowledge gaps. Previous reviews of intervention effectiveness have identified a lack of credible evidence for how to manage direct negative encounters with carnivores [34], or insufficient evidence to draw conclusions about their effect in specifically reducing attacks on people [30]. Instead, a majority of previous reviews have largely focused upon interventions to reduce predation from large carnivores on livestock (e.g., [18, 21, 38, 49]). Although there might be some overlap with regards to the interventions used to prevent attacks from large carnivores on livestock with interventions intended to prevent attacks on people, we are expecting that some interventions may differ. For instance, interventions to prevent attacks on people may need to be portable when people visit carnivore areas, such as some deterrents (e.g., [52]) or could focus on educational efforts (e.g., [3, 5]) or alert systems (e.g., [16]). A review focusing specifically on reducing the direct negative impacts of large carnivores on humans, while including all possible evaluated interventions is still lacking.

In this protocol and the attached ROSES form (Additional file 1) we outline a systematic review of evidence for intervention effectiveness to reduce the risk or severity of direct attacks on humans by large carnivores. Specifically, the review focuses on articles with focal wild species that are native to the study areas, as invasive or non-native species may be subject to a different management scheme than native fauna. For instance, whereas non-native species may be subject to eradication programs locally (e.g., [35, 39]), other means of reducing the negative impacts caused by carnivores of conservation concern are necessary. If wildlife managers and conservationists, as well as other people who may interact with large carnivores, would have accessible knowledge of available interventions and their effectiveness, then improvement in the recommendations can be made regarding the most appropriate interventions and its correct implementation, leading to a decrease in risky encounters between people and large carnivores. This is the aim of the systematic review described in this protocol. The protocol has been registered in PROCEED (https://www.proceedevidence.info/site/index) prior to publication in Environmental Evidence, manuscript number PROCEED-24-00227.

The review is commissioned by the Wildlife Damage Center at the Swedish University of Agricultural Sciences and is funded by the EU project LIFE—Wild Wolf (LIFE21-NAT-IT-LIFE WILD WOLF). SWDC representatives are engaged in the review work group and introduced the research question in addition to determine the scope and focus. As coauthors on the project, SWDC representatives have worked on developing the search strategy and search string. Collaboration is continuous throughout every part of the review process, and feedback provided through written communication alongside multiple workgroup meetings.

## Objective of the review

**Fig. 1:**
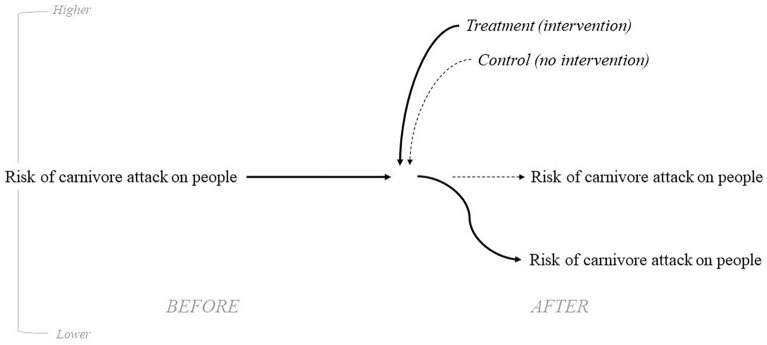
Causal diagram illustrating how treatment settings are assumed to reduce the risk of carnivore attacks (solid lines) while the risk is assumed to remain constant in the control settings (dashed lines)

The review seeks to answer the following question:

- How effective are evaluated interventions in reducing the prevalence of large carnivores near humas and/or attacks on people? (Fig. 1)

## Methods

### Searching for articles

Three bibliographic databases will be searched using the search string in Table A3.1 (supplementary material 3, search string), these are Zoological Record, BIOSIS Citation Index, and Scopus. Literature searches in bibliographical databases are undertaken with the subscriptions of the Swedish University of Agricultural Sciences. The search string is composed of five search term categories based on the P-I-C-O (Population, Intervention, Comparator, Outcome) elements, which are described in detail under *Eligibility Criteria*. The different criteria are target (Population), counteraction (Intervention), evaluation (Comparator) and species/behavior (Outcome). Target terms were added by the work group, aiming to capture synonyms to humans and anthropogenic resources that may attract large carnivores and lead to human-carnivore interactions. Species terms include the common names of terrestrial large carnivore species, i.e., carnivores of > 15 kg body mass, [45], and coyotes which can have a body mass of < 15 kg but are known to sometimes impact human health in part of their range (e.g., [51]). Behavior and counteraction terms were initially added by the work group, but additional terms were added informed by the benchmark articles (Additional file 2). Evaluation terms were added by the work group and amended through scoping of benchmark articles.

A topic search (TS =) will be made in Zoological Record and BIOSIS Citation Index using the Web of Science search engine (exact search option). The topic search includes a search of titles and abstracts alongside other descriptors. Initial scoping searches were undertaken in Web of Science (all databases) and in Scopus. A set of twelve benchmark articles (Additional file 2) were used to evaluate the ability of the search string to return relevant articles from the databases. Seven benchmark articles were gathered from the research portal Conservation Evidence (https://www.conservationevidence.com/) where actions were searched with the keywords “attack” and “safety”. Returned references under the topics “*Use non-lethal methods to deter carnivores from attacking humans*”, “*Translocate problem mammals away from residential areas (e.g. habituated bears) to reduce human-wildlife conflict*”, “*Provide education programmes to improve behaviour towards mammals and reduce threats*”, and “*Scare or otherwise deter mammals from human-occupied areas to reduce human-wildlife conflict*” were screened according to the review eligibility criteria. The additional five benchmark articles were added by the authors.

The benchmark articles informed amendments of the search string by adding terms to the search categories. The amended and final search string generated the largest return (11 of 12 benchmark articles) from Zoological Record, BIOSIS Citation Index, and Scopus. The total number of returned titles during scoping searches were 12,147 in Zoological Record and BIOSIS Citation Index alone, while including searches in CABI abstracts and Web of Science Core Collection returned 25,201 titles. We therefore decided that, due to resource constraints, the search in Web of Science would be limited to Zoological Record and BIOSIS Citation Index. Scoping searches in Scopus were limited to relevant topic searches and returned 5444 titles, including 9 benchmark articles. Searches in Scopus were complementary to the Web of Science searches, despite returning a smaller number of benchmark articles in total. The missing benchmark article [37] was unavailable in all databases subjected to scoping searches. The final search terms and search strings are shown in Additional file 3.

To search for grey literature and unpublished studies, we will search organisational websites and contact experts within the field to share a request for articles within their networks. The digital collection of USDA Wildlife Services (https://nwrc.contentdm.oclc.org/digital/collection/NWRCPubs1/search), Norwegian Institute for Nature Research (NINA, https://www.nina.no/english/Publications), Wildlife Institute of India (https://wii.gov.in/) will be searched using population and intervention search terms. Outreach and requests for studies will be made via colleagues and previous collaborators, and through contacts in main conservation organizations (including but not limited to Panthera https://panthera.org/, African Wildlife Foundation https://www.awf.org/, Wildlife Conservation Society https://www.wcs.org/, Wildlife Conservation Network https://wildnet.org/, and Bear Smart https://www.bearsmart.com/) that focus at least in part on mitigation of wildlife conflicts. Requests for studies may also be posted in relevant social media.

We expect that the search will return a number of prior review articles, which will not be included in the final set of studies but will be screened for original research articles in their references. The reference lists of the final set of articles will also be screened, as will articles that have cited included articles, using the citation search in Web of Science. If the time and budget allow a search update, an updated search may be performed after the final screening.

### Article screening and study eligibility

#### Screening process

Titles and abstracts returned from the database searches will be imported to an online Rayyan (https://www.rayyan.ai/) account. Approximately 22,000 titles are expected to be returned from the search in Zoological Record, BIOSIS Citation Index, and Scopus. Exact duplicates will be automatically removed on the import into Rayyan. We will then use the “detect duplicates” function to find further potential duplicates among the titles. All potential duplicates will be manually screened and verified duplicates will be removed from the set.

The remaining articles will undergo manual screening in two steps. First, all returned titles and abstracts will be screened for including eligible population and intervention. This screening of titles and abstracts will be undertaken in Rayyan, mainly by one screener. For consistency, a minimum of 5% of the titles/abstracts will be screened by a second screener, and Cohen’s Kappa will be calculated. All disagreements will be resolved by discussion. Bibliographic information (title, author, publication year, journal) of all articles retained for full-text reading (including relevant studies or studies which cannot be determined as irrelevant because sufficient information is missing in the title and abstract) will be recorded under the “ELIGIBILITY” tab in the supplementary Excel data sheet (Additional file 4). Irrelevant articles will be excluded from further analysis.

The second step concerns any publication that is not excluded in the first screening step, each of which will be read in full. During the full text reading, publication eligibility will be determined with regards to inclusion of a relevant population, intervention, comparator, and outcome (see eligibility criteria). Eligibility for population, intervention, comparator, and outcome will be coded yes/no/unclear in the ELIGIBILITY tab in the datasheet (Additional file 4). Only articles for which all the eligibility criteria are met (coded “yes”) will be retained for analysis and synthesis. Articles for which one or more criteria are not met (coded “no”) will be excluded. If eligibility is unclear, for instance because insufficient information is provided in the text, the authors of the original article will be contacted for more information and detail. Any author communications will be recorded in Additional file 5. The records (Additional file 5) include information about the date, message, and study id-number. For any publication excluded during eligibility assessment, extracted bibliographic information will remain in the datasheet along with the stated reason for exclusion. Any detected and suspected linkages between articles will also be noted in the datasheet. Where linkages are not clear from full text reading, authors of the original article will be contacted to confirm or dismiss the link, and communications recorded in Additional file 5. Data from linked (or suspectedly linked) articles will be handled with consideration to overlaps, to avoid double counting, and linkages will be noted in the ELIGIBILITY data sheet as well as be reported in the syntheses.

The second step screening, including the main part of full text reading, will be undertaken by one reviewer. However, a random sample of at least 5% of the articles will be screened in parallel by a second reviewer for consistency checking. Consistency will be estimated through calculation of Cohen’s Kappa coefficient. Where disagreements occur, these will be discussed until consensus is reached. Reviewers who appear as authors of original papers will not review their own work, nor undertake eligibility assessment and validity judgments of the publication. In instances where the reviewers are unable to reach consensus alone, or where reviewers occur as the authors of a publication, another member of the review team will be engaged in settling the disagreement or screen the article. If this is not sufficient, a review panel consisting of additional researchers are available for consultation.

#### Eligibility criteria

Articles eligible for inclusion in the analysis will describe the Population, Intervention, Comparator, and Outcome (PICO) which have shaped the review research question. Eligibility criteria were developed together with the stakeholders to ensure the relevance for them and their funders and were evaluated through eligibility screening of the benchmark articles. Articles included in the review will report studies with the following elements:

1. **Population.** People interacting with large carnivores. In the review context, included large carnivores are wild animals within the order *Carnivora* with a body mass > 15 kg that can pose a direct threat to human safety, and are free-living in the wild (i.e., not captive or tamed). In addition to species listed by Ripple et al. [45] we also include coyotes (*Canis latrans*) as the species matches the definition within parts of the range.
2. **Intervention.** Any method, action, or technology implemented to reduce the likelihood of risky encounters between large carnivores and people, or attacks from large carnivores on people.
3. **Comparator.** Intervention/control comparison where at least one treatment (exposure to focal intervention) setting is compared to at least one control (no exposure to focal intervention) setting. If additional interventions are undertaken in the control setting these must also be undertaken alongside the focal intervention in the treatment setting, to meet the criteria.
4. **Outcome.** Quantitative measures and comparisons of the prevalence of large carnivores in or near human settlement, the occurrence/intensity of close encounters between large carnivores and people, changes in flight initiation distance of carnivores before/after treatment, or attacks on people, in the treatment and control settings (i.e., evaluations of intervention effectiveness).

Because of the language limitations of the review team, included articles must be written in English, Spanish, or Swedish. Only original studies will be eligible for inclusion whether published as scientific articles, books chapters, proceeding etc. A list of all articles excluded at full-text reading, and the reason for their exclusion, will be provided.

### Study validity assessment

**Fig. 2:**
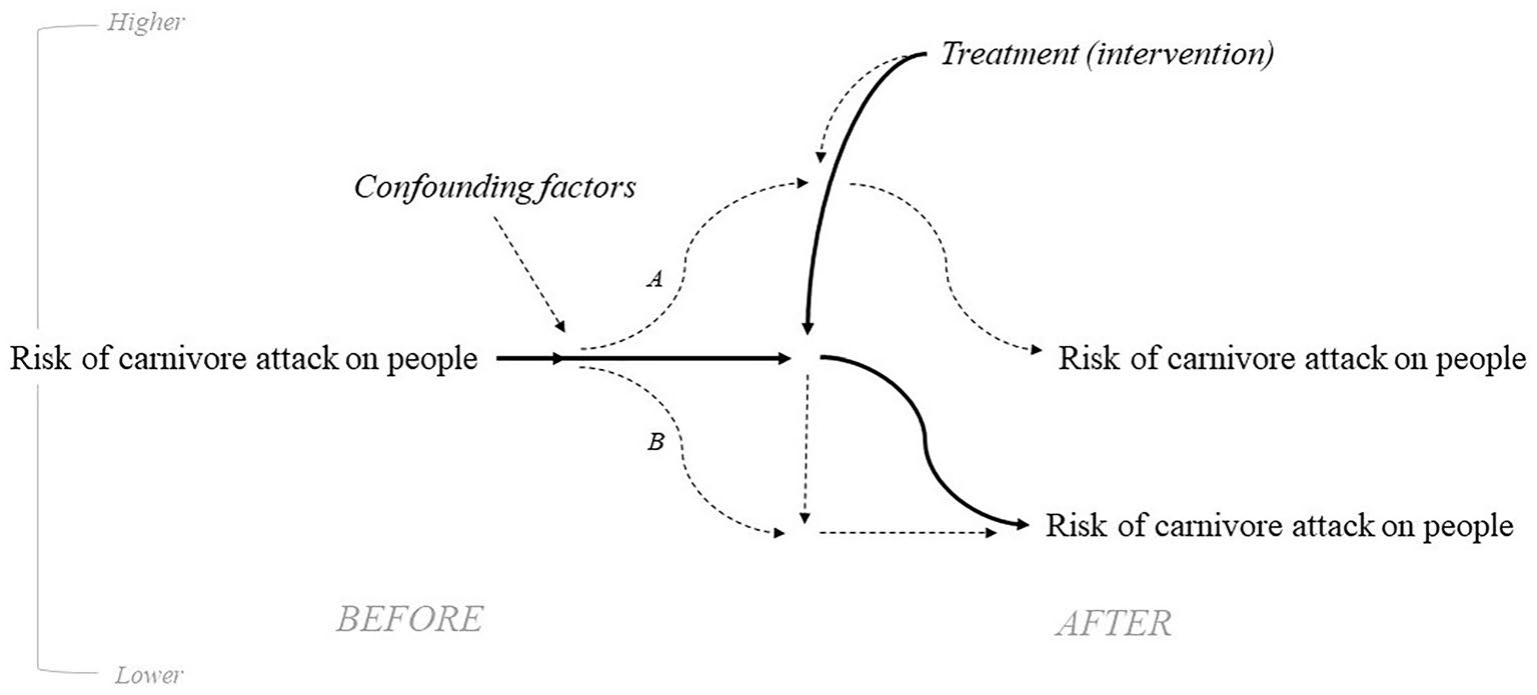
Causal diagram illustrating how confounding factors (dashed lines) could interfere with the assumed causal relationship between intervention and outcome (solid line) and complicate the interpretation of measured outcomes. Scenario A describes how confounding factors in the treatment setting have increased the risk of carnivore attack, and even if the intervention reduces the risk this effect of the intervention is obscured. Scenario B describes how confounding factors in the treatment setting have reduced the risk of carnivore attack prior to implementation of the intervention, and the ineffectiveness of the intervention to further reduce the risk is obscured

Critical appraisal of study validity in the included studies will be undertaken by two reviewers, using the Collaboration for Environmental Evidence Critical Appraisal Tool prototype version 0.3, which is specifically developed for critical appraisal of studies within environmental research [31]. Disagreements about judgments will be discussed until consensus is reached, or else a third reviewer will be invited to perform an additional critical appraisal of the study and settle the disagreement. Risk of compromised internal validity in the included studies will be appraised according to the tool’s seven criteria: 1. risk of confounding biases, 2. risk of post-intervention selection biases, 3. risk of misclassified comparison biases (observational studies only), 4. risk of performance biases (experimental studies only), 5. risk of detection biases, 6. risk of outcome reporting biases, and 7. risk of outcome assessment biases [31]. Potential confounding factors can obscure the effectiveness, or ineffectiveness, of an intervention (Fig. 2) if differences occur between treatment and control settings. Examples of confounding factors may be the presence/absence of domestic animals near people or people’s homes, human behavior, proximity to natural habitat, the contextual motivation of carnivores to attack people (e.g., level of starvation behind predation motivation, importance of resource behind defense motivation, human provocation), or behavioral differences between carnivores (e.g., varying sensitization or habituation to human presence as a consequence of prior treatment etc.). Records of judgments for each of the included studies, with responses to each of the tool’s questions, will be listed in a decisions sheet and the overall bias judgements included in the data sheet (Additional file 4). In the syntheses the judgements will be presented in a table along with concise textual judgement justifications.

### Data coding and extraction strategy

All records of data coding and extraction are documented in a purposely developed data sheet (Additional file 4), based on a data sheet used in a parallel review conducted by an overlapping review team [23]. A pilot test of data extraction in the data sheet was conducted by two reviewers using benchmark articles. Additional file 4 includes several tabs. Data extraction is undertaken under the tab ANALYSIS, where data is linked to the original article through a study id provided by the reviewers, and which is identical to the study id under the ELIGIBILITY tab. A detailed description for data extraction is provided under the tab “CODING INSTRUCTION”. Extracted data include records of study context e.g., geographic location, large carnivore species, and intervention type and specifics etc.), experiment detail (e.g., duration of study, statistical unit etc.), and effect estimates (e.g., sample size, effect measures). Some studies are expected to report their results in figures rather than providing exact numbers for measured effects, and in these cases the online tool PlotDigitizer (https://plotdigitizer.com/app) will be employed to extract values. Two reviewers will extract values using the tool, and potential challenges of using the software will be consider, to enhance the accuracy of value extraction [4]. It is also expected that studies can lack sufficient reports of effect measures. In this instance, the authors of the original article will be contacted by the review team with a request for data. Communications are recorded in Additional file 5. If authors do not respond, or if they are unable to share the missing data with the review team, the study will be removed from further analysis, and the reason will be stated in the data extraction sheet. This file and the extracted data file, in machine-readable and human-readable formats, will be made available on publication of the final review report.

### Potential effect modifiers/reasons for heterogeneity

Heterogeneity is expected among studies, both due to variation in research designs, but also through contextual effect modifiers. Representatives of the SWDC were consulted, in their role as stakeholders and experts on the topic, to list potential sources of heterogeneity. First, it is expected that, even though large carnivores share certain traits (such as their diet) between species, some species-specific traits (including a diversity of behavioral or physical adaptations) will occur. As an example, a previous review of interventions intended to prevent attacks on livestock differences in intervention effectiveness between carnivores that dig (into enclosures) compared to those that climb [21]. During the analysis care will also be taken with regards to the object subjected to carnivore attacks, e.g., trash vs. humans, as these targets could represent different situations to large carnivores.

Intervention effect may also vary with discrepancies in their implementation or maintenance [24]. Within intervention categories we may also expect different types and designs between studies, i.e., different types of fencing or scaring approaches [21]. Potential heterogeneity stemming from discrepancies in intervention implementation and design, will be considered in the analysis and discussed in the final report. Finally, various biological factors (e.g., gender, age, or reproductive status) and behavior of individual animals could also be expected effect modifiers, but these are difficult to identify in the review analysis. Nevertheless, the potential influence of individual states and traits may be discussed in the review report.

### Data synthesis and presentation

Review outcomes will be synthesized in a narrative, and if possible, a quantitative synthesis. Extracted and coded data will be made available in machine-readable and human-readable formats on publication of the final review. The narrative synthesis will provide reference to all included articles, and describe in text the carnivore population (species, location), context (target object, intervention model), as well as the design and reported results of each study. Narrative presentations will be made based on the intervention category specified in the data extraction sheet. Visualization of the review outcomes will also be made through a diagram of intervention effect in each original study, and a map may be provided over the geographical distribution of studies, providing reference to the original studies, focal species, and intervention category.

The quantitative synthesis will include a summary statistic, preferably logarithmic risk ratio, calculated for each original study. The risk ratio will be calculated as the ratio of the probability of attacks on humans (alternatively carnivore intrusion to settlements or other areas with high risk of encounters) between the treatment and the control setting. It is likely that some studies report dichotomous outcomes while others report continuous outcomes in their results. By recalculating the outcomes as risk ratios, it will be possible to compare the outcomes of different studies. If studies are reporting outcomes as count data, this will be dichotomised prior to the risk ratio calculation. The drawback of using risk rations is that when original studies report continuous outcomes, the conversion to a relative measure (such as the risk ratio) implies a loss of information [15]. To minimize this drawback in the review, we will additionally calculate a standardized mean difference for comparisons between such studies. Provided that enough data is available and that it complies with its assumptions, a meta-regression analysis will be undertaken using metafor package in R [50]. In the case that meta-regression is not possible, original studies will be grouped according to similarities (e.g., intervention category) and the summary statistics presented jointly in tables and figures. A forest plot will be created to visualize study outcomes, as well as judgments of critical appraisal [15].

To identify potential publication bias, a funnel plot where, for each study, the effect measure plotted against the standard error of the effect measure. Provided that grey literature is obtained during the review, the outcomes of these studies may be analyzed in contrast to the scientifically published studies and using the Egger test [20], asymmetry may be detected. Possible causes of the asymmetry and potential publication bias will be discussed [41].

## Supplementary Information

Supplementary Material 1.

Supplementary Material 2.

Supplementary Material 3.

Supplementary Material 4.

Supplementary Material 5.

## Data Availability

All data generated or analysed during this study are included in the published article [and its supplementary information files].

## References

[1] Acharya KP, Paudel PK, Neupane PR, Köhl M. Human-wildlife conflicts in Nepal: patterns of human fatalities and injuries caused by large mammals. PLoS ONE. 2016;11: e0161717. 27612174 10.1371/journal.pone.0161717 PMC5017643

[2] Alldredge MW, Walsh DP, Sweanor LL, Davies RB, Trujillo A. Evaluation of translocation of black bears involved in human-bear conflicts in South-Central Colorado. Wildlife Soc B. 2015;39:334–40. 10.1002/wsb.526

[3] Appleby R, Smith B, Mackie J, Bemede L, Jones D. Preliminary observations of dingo responses to assumed aversive stimuli. Pac Conserv Biol. 2017;23:295–301. 10.1071/PC17005

[4] Aydin O, Yassikaya MY. Validity and reliability analysis of the PlotDigitzer software program for data extraction from single-case graphs. Perspect Behav Sci. 2022;45:239–57. 35342869 10.1007/s40614-021-00284-0 PMC8894524

[5] Baruch-Mordo S, Breck SW, Wilson KR, Broderick J. The carrot or the stick? Evaluation of education and enforcement as management tools for human-wildlife conflicts. PLoS ONE. 2011;6: e15681. 21264267 10.1371/journal.pone.0015681 PMC3020222

[6] Bombieri G, et al. Brown bear attacks on humans: a worldwide perspective. Sci Rep-UK. 2019;9:8573. 10.1038/s41598-019-44341-w PMC6562097 31189927

[7] Bombieri G, et al. A worldwide perspective on large carnivore attacks on humans. PLoS Biol. 2023;21: e3001946. 36719873 10.1371/journal.pbio.3001946 PMC9888692

[8] Borlaug N. Feeding a hungry world. Science. 2007;318:359–359. 17947551 10.1126/science.1151062

[9] Carter NH, Linnell JD. Co-adaptation is key to coexisting with large carnivores. Trends Ecol Evol. 2016;31:575–8. 27377600 10.1016/j.tree.2016.05.006

[10] Chapron G, et al. Recovery of large carnivores in Europe’s modern human-dominated landscapes. Science. 2014;346:1517–9. 25525247 10.1126/science.1257553

[11] Chowdhury AN, Brahma A, Mondal R, Biswas MK. Stigma of tiger attack: study of tiger-widows from Sundarban Delta, India. Indian J Psychiat. 2016;58:12–9. 10.4103/0019-5545.174355 PMC4776575 26985099

[12] Conover MR. Numbers of human fatalities, injuries, and illnesses in the United States due to wildlife. Hum-Wildl Interact. 2019;13:264–76.

[13] Crespin SJ, Simonetti JA. Reconciling farming and wild nature: Integrating human–wildlife coexistence into the land-sharing and land-sparing framework. Ambio. 2019;48:131–8. 29752683 10.1007/s13280-018-1059-2 PMC6346604

[14] Davies JC, Rockwell RF. An electric fence to deter polar bears. Wildlife Soc B. 1986;14:406–406.

[15] Deeks JJ, Higgins JPT, Altman DG. Chapter 10: Analysing data and undertaking meta-analyses. In: Higgins JPT, Thomas J, Chandler J, Cumpston M, Li T, Page MJ, Welch VA (editors). Cochrane Handbook for Systematic Reviews of Interventions version 6.3 (updated February 2022). Cochrane. 2022. www.training.cochrane.org/handbook. Accessed 10 Sept 2023.

[16] Dertien JS, et al. Mitigating human–wildlife conflict and monitoring endangered tigers using a real-time camera-based alert system. Bioscience. 2023;73:748–57. 37854891 10.1093/biosci/biad076 PMC10580963

[17] Dhar SA, Butt MF, Farooq M, Mir MR, Wani ZA, Afzal S, Sultan A, Wani MI. Pattern of orthopaedic injuries in bear attacks: report from a tertiary care centre in Kashmir. Injury. 2008;39:249–55. 18093590 10.1016/j.injury.2007.07.028

[18] van Eeden L, et al. Carnivore conservation needs evidence-based livestock protection. PLoS Biol. 2018;16:e2005577. 30226872 10.1371/journal.pbio.2005577 PMC6143182

[19] Edgar JP, Appleby RG, Jones DN. Efficacy of an ultrasonic device as a deterrent to dingoes (Canis lupus dingo): a preliminary investigation. J Ethol. 2007;25:209–13. 10.1007/s10164-006-0004-1

[20] Egger M, Smith GD, Schneider M, Minder C. Bias in meta-analysis detected by a simple, graphical test. BMJ. 1997;315:629. 9310563 10.1136/bmj.315.7109.629 PMC2127453

[21] Eklund A, López-Bao JV, Tourani M, Chapron G, Frank J. Limited evidence on the effectiveness of interventions to reduce livestock predation by large carnivores. Sci Rep-UK. 2017;7:2097. 10.1038/s41598-017-02323-w PMC5437004 28522834

[22] Eklund A, Månsson J, Frank J. How effective are interventions to reduce damage to agricultural crops from herbivorous wild birds and mammals? A systematic review protocol. Environ Evid. 2023;12:22. 10.1186/s13750-023-00315-0 PMC11378812 39294803

[23] Eklund A, Waldo Å, Johansson M, Frank J. Navigating, “Human Wildlife Conflict” situations from the individual’s perspective. Biol Conserv. 2023;283: 110117. 10.1016/j.biocon.2023.110117

[24] Frank J, Eklund A. Poor construction, not time, takes its toll on subsidised fences designed to deter large carnivores. PLoS ONE. 2017;12: e0175211. 28394912 10.1371/journal.pone.0175211 PMC5386237

[25] Green RE, et al. Farming and the fate of wild nature. Science. 2005;307:550–5. 15618485 10.1126/science.1106049

[26] Herrero S, Higgins A. Field use of capsicum spray as a bear deterrent. Ursus. 1998;10:533–7.

[27] Jhala YV, Qureshi Q, Nayak AK. (eds). Status of tigers, co-predators and prey in India 2018. Summary Report. National Tiger Conservation Authority, Government of India, New Delhi & Wildlife Institute of India, Dehradun. 2019;TR No./2019/05.

[28] Jope KL. Implications of Grizzly Bear Habituation to Hikers. Wildlife Soc B. 1985;13:32–7.

[29] Keesing F, Ostfeld RS. Impacts of biodiversity and biodiversity loss on zoonotic diseases. PNAS. 2021;118: e2023540118. 33820825 10.1073/pnas.2023540118 PMC8092607

[30] Khorozyan I, Waltert M. A framework of most effective practices in protecting human assets from predators. Hum Dimens Wildl. 2019;24:380–94. 10.1080/10871209.2019.1619883

[31] Konno K, Livoreil B, Pullin AS. Collaboration for Environmental Evidence Critical Appraisal Tool version 0.3 (prototype). Collaboration for Environmental Evidence. 2021. https://environmentalevidence.org/cee-critical-appraisal-tool/. Accessed 20 Sept 2023.

[32] Linnell JDC, Kovtun E, Rouart I. Wolf attacks on humans: an update for 2002–2020. Norwegian Institute for Nature Research. 2021;NINA Report 1944.

[33] López-Bao JV, et al. Carnivore coexistence: wilderness not required. Science. 2015;348:871–2. 10.1126/science.348.6237.871-b 25999497

[34] Löe J, Röskaft E. Large carnivores and human safety: a review. Ambio. 2004;33:283–8. 15387060 10.1579/0044-7447-33.6.283

[35] Mazzamuto MV, Panzeri M, Bisi F, Wauters LA, Preatoni D, Martinoli A. When management meets science: adaptive analysis for the optimization of the eradication of the Northern raccoon (Procyon lotor). Biol Invasions. 2020;22:3119–30. 10.1007/s10530-020-02313-6

[36] Methorst J, Arbieu U, Bonn A, Böhning-Gaese K, Müller T. Non-material contributions of wildlife to human well-being: a systematic review. Environ Res Lett. 2020;15: 093005. 10.1088/1748-9326/ab9927

[37] Miller GD. Field tests of potential polar bear repellents. Int C Bear. 1986;7:383–90.

[38] Miller JRB, Stoner KJ, Cejtin MR, Meyer TK, Middleton AD, Schmitz OJ. Effectiveness of contemporary techniques for reducing livestock depredations by large carnivores. Wildlife Soc B. 2016;40:806–15. 10.1002/wsb.720

[39] Moore NP, Roy SS, Helyar A. Mink (Mustela vison) eradication to protect ground-nesting birds in the Western Isles, Scotland, United Kingdom. New Zeal J Zool. 2003;30:443–52. 10.1080/03014223.2003.9518351

[40] Packer C, et al. Species-specific spatiotemporal patterns of leopard, lion and tiger attacks on humans. J Appl Ecol. 2018;56:585–93. 10.1111/1365-2664.13311

[41] Page MJ, Higgins JPT, Sterne JAC. Chapter 13: Assessing risk of bias due to missing results in a synthesis. In: Higgins JPT, Thomas J, Chandler J, Cumpston M, Li T, Page MJ, Welch VA (editors). Cochrane Handbook for Systematic Reviews of Interventions version 6.3 (updated February 2022). Cochrane. 2022. www.training.cochrane.org/handbook. Accessed 10 Sept 2023.

[42] Penteriani V, et al. Human behaviour can trigger large carnivore attacks in developed countries. Sci Rep. 2016;6:20552. 26838467 10.1038/srep20552 PMC4738333

[43] Phalan B, et al. Reconciling food production and biodiversity conservation: land sharing and land sparing compared. Science. 2011;333:1289–91. 21885781 10.1126/science.1208742

[44] Ratnayeke S, Van Manen FT, Pieris R, Pragash VSJ. Challenges of large carnivore conservation: sloth bear attacks in Sri Lanka. Hum Ecol. 2014;42:467–79.

[45] Ripple WJ, et al. Status and ecological effects of the world’s largest carnivores. Science. 2014;343:1241484. 24408439 10.1126/science.1241484

[46] Santini L, et al. Incorporating spatial population structure in gap analysis reveals inequitable assessments of species protection. Divers Dist. 2014;20:698. 10.1111/ddi.12198

[47] Sponarski CC, Vaske JJ, Bath AJ, Loeffler TA. Changing attitudes and emotions toward coyotes with experiential educationet. J Environ Educ. 2016. 10.1080/00958964.2016.1158142. 10.1080/00958964.2016.1158142

[48] Treves A, Karanth KU. Human-carnivore conflict and perspectives on carnivore management worldwide. Conserv Biol. 2003;17:1491–9. 10.1111/j.1523-1739.2003.00059.x

[49] Treves A, Krofel M, McManus J. Predator control should not be a shot in the dark. Front Ecol Environ. 2016;14:380–8. 10.1002/fee.1312

[50] Viechtbauer W. Conducting meta-analyses in R with the metafor package. J Stat Softw. 2010;36:1–48. 10.18637/jss.v036.i03. 10.18637/jss.v036.i03

[51] White LA, Gehrt SD. Coyote attacks on humans in the United States and Canada. Hum Dimens Wildl. 2009;14:419–32. 10.1080/10871200903055326

[52] Wilder JM, et al. Efficacy of bear spray as a deterrent against polar bears. Wildlife Soc B. 2023;47:e1403. 10.1002/wsb.1403

