# Supplementary material for: How effective are interventions to reduce attacks on people from large carnivores? A systematic review protocol
Source: Environ Evid. 2024 May 17;13:13. doi: 10.1186/s13750-024-00337-2 (PMC11378859; doi:10.1186/s13750-024-00337-2)
Supplement: Supplementary file 2 — Supplementary Material 2. [file 13750_2024_337_MOESM2_ESM.docx]

**Benchmark articles**

**Benchmark articles from search in Conservation Evidence**

References derived from Conservation Evidence (<https://www.conservationevidence.com/>) where actions were searched with the keywords “attack” and “safety”. Eligible references were returned under the topics “*Use non-lethal methods to deter carnivores from attacking humans*”, “*Translocate problem mammals away from residential areas (e.g. habituated bears) to reduce human-wildlife conflict*”, “*Provide education programmes to improve behaviour towards mammals and reduce threats*”, and “*Scare or otherwise deter mammals from human-occupied areas to reduce human-wildlife conflict*”.

1. Appleby R, Smith B, Mackie J, Bernede L, Jones D. Preliminary observations of dingo responses to assumed aversive stimuli. Pac Conserv Biol. 2017;23:295-301.
2. Baruch-Mordo S, Berck SW, Wilson KR, Broderick J. The carrot or the stick? Evaluation of education and enforcement as management tools for human-wildlife conflicts. PLoS ONE 2011;DOI:10.1371/journal.pone.0015681.
3. Beckmann JP, Lackey CW, Berger J. Deterrent techniques and dogs to alter behavior of “nuisance” black bears. Wildlife Soc B. 2004;32:1141-1146.
4. Breck SW, Lance N, Callahan P. Shocking device for protection of concentrated food sources from black bears. Wildlife Soc B 2006;34:23-26.
5. Breck SW, Poessel SA, Bonnell MA. Evaluating lethal and nonlethal management options for urban coyotes. Hum Wildlife Interact. 2017;11:133-145.
6. Miller GD. Field tests of potential polar bear repellents. Bears: Their Biology and Management, Vol. 7, A Selection of Papers from the Seventh International Conference on Bear Research and Management, Williamsburg, Virginia, USA, and Plitvice Lakes, Yugoslavia. 1986:383-390.
7. Pop IM, Sallay A, Bereczky L, Chiriac S. Land use and behavioral patterns of brown bears in the South-Eastern Romanian Carpathian Mountains: A case study relocated and rehabilitated individuals. Procedia Environm Sci. 2012;14:111-122.

**Benchmark articles added by the author team**

1. McLellan & Walker. Efficacy of motion-activated sprinklers as a humane deterrent for urban coyotes. Hum Dim Wildl. 2021;26:76-83.
2. Northrup, Howe, Inglis, Newton, Obbard, Pond, Potter. Experimental test of the efficacy of hunting for controlling human–wildlife conflict. J Wildl Manage. 2022;DOI: 10.1002/jwmg.22363.
3. Sarmento. Bear deterrence with scare devices, a non-lethal tool in the use-of-force continuum. J Wildl Manage. 2024;DOI: 10.1002/jwmg.22552.
4. Smith, Herrero, Debruyn, Wilder. Efficacy of Bear Deterrent Spray in Alaska. J Wild Manage. 2008;72: DOI: 10.2193/2006-452.
5. Young & Sarmento. Can an old dog learn a new trick? : Efficacy of livestock guardian dogs at keeping an apex predator away from people. Biol Conserv. 2024;292:110554.
