# Supplementary material for: How effective are interventions to reduce attacks on people from large carnivores? A systematic review protocol
Source: Environ Evid. 2024 May 17;13:13. doi: 10.1186/s13750-024-00337-2 (PMC11378859; doi:10.1186/s13750-024-00337-2)
Supplement: Supplementary file 3 — Supplementary Material 3. [file 13750_2024_337_MOESM3_ESM.docx]

**Search string**

Table A3.1. Search terms, search categories, and search strings for Web of Science and Scopus searches. In Web of Science the “___” define exact compound terms and the * is used as a wildcard that can replace any number of characters, or no characters. In Scopus searches the {___} is used for exact compound terms.

| *Population* | *Intervention* | *Comparator* | *Outcome* | |
| --- | --- | --- | --- | --- |
| **TARGET** | **COUNTERACTION** | **EVALUATION** | **SPECIES** | **BEHAVIOUR** |
| human* people public man men woman women child  children worker* victim*  waste trash bin (anthropogenic food source) bait*  residential  urban | protect* prevent* mitigat* manag* intervention* action* remov* repel* scare scaring displace displacing haze hazing conditioning divert* deter* barrier* hunt* buffer zone buffer zones translocation* guard* warn* noise* | trial*  experiment*  evaluat*  effect* test* efficacy  evidence | large carnivore*  wolf  wolves  African wild dog* dhole* dingo* tiger*  lion* jaguar* cheetah* leopard* puma* mountain lion* Florida panther*  cougar* lynx  bear* giant panda  hyena* wolverine*  coyote* | predat* attack* kill* injur* maraud* fatal* safety threat* nuisance  encounter*  conflict* visit*  intrusion intrude |
| *Web of Science search (Zoological Record & BIOSIS Citation Index)*  TS= ((human* OR people OR public OR man OR men OR woman OR women OR child OR children OR worker* OR victim* OR waste OR trash OR bin OR “anthropogenic food source” OR bait* OR residential OR urban) AND (protect* OR prevent* OR mitigat* OR manag* OR intervention* OR action* OR remov* OR repel* OR scare OR scaring OR displace OR displacing OR haze OR hazing OR conditioning OR divert* OR deter* OR barrier* OR hunt* OR “buffer zone*” OR translocation* OR guard* OR warn* OR noise*) AND (trial* OR experiment* OR evaluat* OR effect* OR test* OR efficacy OR evidence)  AND ((“large carnivore*” OR wolf OR wolves OR “African wild dog*” OR dhole* OR dingo* OR tiger* OR lion* OR jaguar* OR cheetah* OR leopard* OR puma* OR “mountain lion*” OR “Florida panther*” OR cougar* OR lynx OR bear* OR giant panda OR hyena* OR wolverine* OR coyote*) AND (predat* OR attack* OR kill* OR injur* OR maraud* OR fatal* OR safety OR threat* OR nuisance OR encounter* OR conflict* OR visit* OR intrude OR intrusion))) | | | | |
| *Scopus search*  TITLE-ABS-KEY= ((human* OR people OR public OR man OR men OR woman OR women OR child OR children OR worker* OR victim* OR waste OR trash OR bin OR {anthropogenic food source} OR bait* OR residential OR urban) AND (protect* OR prevent* OR mitigat* OR manag* OR intervention* OR action* OR remov* OR repel* OR scare OR scaring OR displace OR displacing OR haze OR hazing OR conditioning OR divert* OR deter* OR barrier* OR hunt* OR {buffer zone} OR {buffer zones} OR translocation* OR guard* OR warn* OR noise*) AND (trial* OR experiment* OR evaluat* OR effect* OR test* OR efficacy OR evidence) AND (({large carnivore} OR wolf OR wolves OR {African wild dog} OR {African wild dogs} OR dhole* OR dingo* OR tiger* OR lion* OR jaguar* OR cheetah* OR leopard* OR puma* OR {mountain lion} OR {mountain lions} OR {Florida panther} OR {Florida panthers} OR lynx OR bear* OR {giant panda} OR hyena* OR wolverine* OR coyote*) AND (predat* OR attack* OR kill* OR injur* OR maraud* OR fatal* OR safety OR threat* OR nuisance OR encounter* OR conflict* OR visit* OR intrude OR intrusion))) AND ( LIMIT-TO ( SUBJAREA,"AGRI" ) OR LIMIT-TO ( SUBJAREA,"ENVI" ) OR LIMIT-TO ( SUBJAREA,"MULT" ) OR LIMIT-TO ( SUBJAREA,"SOCI" ) OR LIMIT-TO ( SUBJAREA,"EART" ) OR LIMIT-TO ( SUBJAREA,"VETE" ) OR LIMIT-TO ( SUBJAREA,"Undefined" ) OR LIMIT-TO ( SUBJAREA,"PSYC" ) ) AND ( LIMIT-TO ( LANGUAGE,"English" ) ) | | | | |
